# Supplementary material for: Inverse design of nonlinear metasurfaces for sum frequency generation
Source: Nanophotonics. 2024 Jun 5;13(18):3363–72. doi: 10.1515/nanoph-2024-0137 (PMC11501459; doi:10.1515/nanoph-2024-0137)
Supplement: Supplementary file 1 — Supplementary Material Details [file j_nanoph-2024-0137_suppl_001.pdf]

Supplementary Information:
Inverse Design of Nonlinear Metasurfaces for Sum Frequency Generation

Neuton Li,[1,*] Jihua Zhang,[1,2] Dragomir N. Neshev,[1] and Andrey A. Sukhorukov[1]

[1]*ARC Centre of Excellence for Transformative Meta-Optical Systems (TMOS), Department of Electronic Materials Engineering, Research School of Physics, The Australian National University, Canberra, ACT 2600, Australia*

[2]*Songshan Lake Materials Laboratory, Dongguan, Guangdong 523808, P. R. China*

(Dated: May 23, 2024)

This Supporting Information contains 5 sections and 7 figures, providing extra details on the analytical and numerical aspects of our work.

* neuton.li@anu.edu.au

CONTENTS

S1. Nonlinear Adjoint Topology Optimisation S-3

S1.1. General formulation: E and H fields S-3

S1.2. Scattering into Specific Modes S-6

S1.3. Adjoints and Gradients for Optimization of SFG Processes S-8

S1.4. Re-derivation of Maximum Second Harmonic Flux Gradients S-9

S2. Nonlinear Polarisation of InGaP S-10

S3. Nonlinear Scattering Matrix S-10

S3.1. Plain Unpatterned Film S-10

S3.2. Polarising Nonlinear Metasurface S-11

S3.3. Polarization Independent Nonlinear Metasurface S-11

S4. Diffraction Order Ratio S-12

S4.1. Polarising Nonlinear Metasurface S-12

S4.2. Nonlinear Polarization Independent Metasurface S-13

S5. Linear Transmission Simulations S-13

S5.1. Transmission Spectra S-14

S5.2. Field Distributions S-14

S1. NONLINEAR ADJOINT TOPOLOGY OPTIMISATION

In this section, we derive the constituent equations that are required for adjoint topology optimisation of metasurfaces for SFG processes.

We follow Refs. [S1–S6] to formulate the adjoint optimization approach for nonlinear metasurfaces. Previously, nonlinear cavities [S7] and metasurfaces [S8, S9] were optimized to maximize the total second-harmonic generation. It is of interest to optimize for other nonlinear processes including sum frequency generation (SFG). Additionally, optimization for specific radiation directions, rather than just the total conversion, may be important.

S1.1. General formulation: E and H fields

To begin, we desire a quantity that captures the different properties of light that include polarisation, phase, etc.. One such quantity is the complex coefficient of the overlap between the total scattered electromagnetic wave and a specific electromagnetic mode. We note that this mode amplitude is defined through a specific overlap integral of both the electric and magnetic field components [S2]. Therefore, we modify the derivations in Refs. [S3, S7] to use two wave equations that incorporate both the electric and magnetic fields in the form

$$\mathbf{R}_{E,q}(\mathbf{E},\mathbf{H},\mathbf{p}) = \nabla \times \mathbf{H}_q + i\omega_q\varepsilon_q\mathbf{E}_q - \mathbf{J}_q = 0, \tag{S1}$$

$$\mathbf{R}_{H,q}(\mathbf{E},\mathbf{H},\mathbf{p}) = \nabla \times \mathbf{E}_q - i\omega_q\mu_q\mathbf{H}_q + \mathbf{K}_q = 0. \tag{S2}$$

Here q is an index of the wave with frequency ω_q, $\mathbf{p}$ is a set of parameters to be optimized. $\mathbf{J}_q$ and $\mathbf{K}_q$ are the electric and magnetic current densities, respectively.

Consider an objective function $T(\mathbf{E_{1,2,\ldots,q}}, \mathbf{E_{1,2,\ldots,q}}^*, \mathbf{H_{1,2,\ldots,q}}, \mathbf{H_{1,2,\ldots,q}}^*, \mathbf{p})$ which is a function of the electric and magnetic fields at all positions and various optimization parameters. We want to calculate its derivatives versus these parameters. To simplify notation, consider for a moment a

single scalar parameter p,

$$
\begin{aligned}
\frac{dT}{dp} = \frac{\partial T}{\partial p} &+ \sum_q \frac{\partial T}{\partial \mathbf{E}_q}\frac{\partial \mathbf{E}_q}{\partial p} + \sum_q \frac{\partial T}{\partial \mathbf{H}_q}\frac{\partial \mathbf{H}_q}{\partial p} + \sum_q \frac{\partial T}{\partial \mathbf{E}^*_q}\frac{\partial \mathbf{E}^*_q}{\partial p} + \sum_q \frac{\partial T}{\partial \mathbf{H}^*_q}\frac{\partial \mathbf{H}^*_q}{\partial p} \\
&- 1\left[\frac{\text{Unit of } T/p}{W/m^3}\right]\sum_{q'} \mathbf{v}^T_{E,q'}\left(\frac{\partial \mathbf{R}_{E,q'}}{\partial p} + \sum_q \frac{\partial \mathbf{R}_{E,q'}}{\partial \mathbf{E}_q}\frac{\partial \mathbf{E}_q}{\partial p} + \sum_q \frac{\partial \mathbf{R}_{E,q'}}{\partial \mathbf{H}_q}\frac{\partial \mathbf{H}_q}{\partial p}\right. \\
&\qquad\left. + \sum_q \frac{\partial \mathbf{R}_{E,q'}}{\partial \mathbf{E}^*_q}\frac{\partial \mathbf{E}^*_q}{\partial p} + \sum_q \frac{\partial \mathbf{R}_{E,q'}}{\partial \mathbf{H}^*_q}\frac{\partial \mathbf{H}^*_q}{\partial p}\right) \\
&- 1\left[\frac{\text{Unit of } T/p}{W/m^3}\right]\sum_{q'} \mathbf{v}^T_{H,q'}\left(\frac{\partial \mathbf{R}_{H,q'}}{\partial p} + \sum_q \frac{\partial \mathbf{R}_{H,q'}}{\partial \mathbf{E}_q}\frac{\partial \mathbf{E}_q}{\partial p} + \sum_q \frac{\partial \mathbf{R}_{H,q'}}{\partial \mathbf{H}_q}\frac{\partial \mathbf{H}_q}{\partial p}\right. \\
&\qquad\left. + \sum_q \frac{\partial \mathbf{R}_{H,q'}}{\partial \mathbf{E}^*_q}\frac{\partial \mathbf{E}^*_q}{\partial p} + \sum_q \frac{\partial \mathbf{R}_{H,q'}}{\partial \mathbf{H}^*_q}\frac{\partial \mathbf{H}^*_q}{\partial p}\right) \\
&- 1\left[\frac{\text{Unit of } T/p}{W/m^3}\right]\sum_{q'} \mathbf{u}^\dagger_{E,q'}\left(\frac{\partial \mathbf{R}_{E,q'}}{\partial p} + \sum_q \frac{\partial \mathbf{R}_{E,q'}}{\partial \mathbf{E}_q}\frac{\partial \mathbf{E}_q}{\partial p} + \sum_q \frac{\partial \mathbf{R}_{E,q'}}{\partial \mathbf{H}_q}\frac{\partial \mathbf{H}_q}{\partial p}\right. \\
&\qquad\left. + \sum_q \frac{\partial \mathbf{R}_{E,q'}}{\partial \mathbf{E}^*_q}\frac{\partial \mathbf{E}^*_q}{\partial p} + \sum_q \frac{\partial \mathbf{R}_{E,q'}}{\partial \mathbf{H}^*_q}\frac{\partial \mathbf{H}^*_q}{\partial p}\right)^* \\
&+ 1\left[\frac{\text{Unit of } T/p}{W/m^3}\right]\sum_{q'} \mathbf{u}^\dagger_{H,q'}\left(\frac{\partial \mathbf{R}_{H,q'}}{\partial p} + \sum_q \frac{\partial \mathbf{R}_{H,q'}}{\partial \mathbf{E}_q}\frac{\partial \mathbf{E}_q}{\partial p} + \sum_q \frac{\partial \mathbf{R}_{H,q'}}{\partial \mathbf{H}_q}\frac{\partial \mathbf{H}_q}{\partial p}\right. \\
&\qquad\left. + \sum_q \frac{\partial \mathbf{R}_{H,q'}}{\partial \mathbf{E}^*_q}\frac{\partial \mathbf{E}^*_q}{\partial p} + \sum_q \frac{\partial \mathbf{R}_{H,q'}}{\partial \mathbf{H}^*_q}\frac{\partial \mathbf{H}^*_q}{\partial p}\right)^* .
\end{aligned}
\tag{S3}
$$

Here all of the terms with $\mathbf{v}$ and $\mathbf{u}$ multipliers are zero according to Eqs. (S1),(S2), yet their introduction allows for the efficient derivative calculation. Note that $\{\mathbf{v}_E, \mathbf{u}_E\}$ and $\{\mathbf{v}_H, \mathbf{u}_H\}$ have the units of the electric and magnetic fields, respectively. In the following, we drop the units to simplify the notations.

We now demand that the terms $\partial \mathbf{E}_q/\partial p$ and $\partial \mathbf{H}_q/\partial p$ all vanish in Eq. (S3), which happens when $\mathbf{v}$ satisfy the following relations,

$$
\begin{aligned}
-\frac{\partial T}{\partial \mathbf{E}_q} + i\omega_q \mathbf{v}^T_{E,q}\varepsilon_q - \sum_{q'} \mathbf{v}^T_{E,q'}\frac{\partial \mathbf{J}_{q'}}{\partial \mathbf{E}_q} - \sum_{q'} \mathbf{u}^\dagger_{E,q'}\frac{\partial \mathbf{J}^*_{q'}}{\partial \mathbf{E}_q}& \\
+ \mathbf{v}^T_{H,q}\nabla\times + \sum_{q'} \mathbf{v}^T_{H,q'}\frac{\partial \mathbf{K}_{q'}}{\partial \mathbf{E}_q} + \sum_{q'} \mathbf{u}^\dagger_{H,q'}\frac{\partial \mathbf{K}^*_{q'}}{\partial \mathbf{E}_q} &= 0,
\end{aligned}
\tag{S4}
$$

$$
\begin{aligned}
-\frac{\partial T}{\partial \mathbf{H}_q} - i\omega_q \mathbf{v}^T_{H,q}\mu_q - \sum_{q'} \mathbf{v}^T_{E,q'}\frac{\partial \mathbf{J}_{q'}}{\partial \mathbf{H}_q} - \sum_{q'} \mathbf{u}^\dagger_{E,q'}\frac{\partial \mathbf{J}^*_{q'}}{\partial \mathbf{H}_q}& \\
+ \mathbf{v}^T_{E,q}\nabla\times + \sum_{q'} \mathbf{v}^T_{H,q'}\frac{\partial \mathbf{K}_{q'}}{\partial \mathbf{H}_q} + \sum_{q'} \mathbf{u}^\dagger_{H,q'}\frac{\partial \mathbf{K}^*_{q'}}{\partial \mathbf{H}_q} &= 0.
\end{aligned}
\tag{S5}
$$

By performing the transpose of these two equations, we have

$$\nabla \times \mathbf{v}_{H,q} + i\omega_q \varepsilon_q^T \mathbf{v}_{E,q} - \mathbf{J}_{v,q} = 0, \tag{S6}$$

$$\nabla \times \mathbf{v}_{E,q} - i\omega_q \mu_q^T \mathbf{v}_{H,q} + \mathbf{K}_{v,q} = 0. \tag{S7}$$

where

$$\begin{aligned} \mathbf{J}_{v,q} = \left(\frac{\partial T}{\partial \mathbf{E}_q}\right)^T &+ \sum_{q'} \left(\frac{\partial \mathbf{J}_{q'}}{\partial \mathbf{E}_q}\right)^T \mathbf{v}_{E,q'} + \sum_{q'} \left(\frac{\partial \mathbf{J}^*_{q'}}{\partial \mathbf{E}_q}\right)^T \mathbf{u}^*_{E,q'} \\ &- \sum_{q'} \left(\frac{\partial \mathbf{K}_{q'}}{\partial \mathbf{E}_q}\right)^T \mathbf{v}_{H,q'} - \sum_{q'} \left(\frac{\partial \mathbf{K}^*_{q'}}{\partial \mathbf{E}_q}\right)^T \mathbf{u}^*_{H,q'} , \end{aligned} \tag{S8}$$

$$\begin{aligned} \mathbf{K}_{v,q} = -\left(\frac{\partial T}{\partial \mathbf{H}_q}\right)^T &- \sum_{q'} \left(\frac{\partial \mathbf{J}_{q'}}{\partial \mathbf{H}_q}\right)^T \mathbf{v}_{E,q'} - \sum_{q'} \left(\frac{\partial \mathbf{J}^*_{q'}}{\partial \mathbf{H}_q}\right)^T \mathbf{u}^*_{E,q'} \\ &+ \sum_{q'} \left(\frac{\partial \mathbf{K}_{q'}}{\partial \mathbf{H}_q}\right)^T \mathbf{v}_{H,q'} + \sum_{q'} \left(\frac{\partial \mathbf{K}^*_{q'}}{\partial \mathbf{H}_q}\right)^T \mathbf{u}^*_{H,q'} . \end{aligned} \tag{S9}$$

Similarly, for $\mathbf{u}$ we obtain

$$\nabla \times \mathbf{u}_{H,q} + i\omega_q \varepsilon_q^T \mathbf{u}_{E,q} - \mathbf{J}_{u,q} = 0\,, \tag{S10}$$

$$\nabla \times \mathbf{u}_{E,q} - i\omega_q \mu_q^T \mathbf{u}_{H,q} + \mathbf{K}_{u,q} = 0\,. \tag{S11}$$

where

$$\begin{aligned} \mathbf{J}_{u,q} = \left(\frac{\partial T^*}{\partial \mathbf{E}_q}\right)^T &+ \sum_{q'} \left(\frac{\partial \mathbf{J}_{q'}}{\partial \mathbf{E}_q}\right)^T \mathbf{u}_{E,q'} + \sum_{q'} \left(\frac{\partial \mathbf{J}^*_{q'}}{\partial \mathbf{E}_q}\right)^T \mathbf{v}^*_{E,q'} \\ &- \sum_{q'} \left(\frac{\partial \mathbf{K}_{q'}}{\partial \mathbf{E}_q}\right)^T \mathbf{u}_{H,q'} - \sum_{q'} \left(\frac{\partial \mathbf{K}^*_{q'}}{\partial \mathbf{E}_q}\right)^T \mathbf{v}^*_{H,q'} , \end{aligned} \tag{S12}$$

$$\begin{aligned} \mathbf{K}_{u,q} = -\left(\frac{\partial T^*}{\partial \mathbf{H}_q}\right)^T &- \sum_{q'} \left(\frac{\partial \mathbf{J}_{q'}}{\partial \mathbf{H}_q}\right)^T \mathbf{u}_{E,q'} - \sum_{q'} \left(\frac{\partial \mathbf{J}^*_{q'}}{\partial \mathbf{H}_q}\right)^T \mathbf{v}^*_{E,q'} \\ &+ \sum_{q'} \left(\frac{\partial \mathbf{K}_{q'}}{\partial \mathbf{H}_q}\right)^T \mathbf{u}_{H,q'} + \sum_{q'} \left(\frac{\partial \mathbf{K}^*_{q'}}{\partial \mathbf{H}_q}\right)^T \mathbf{v}^*_{H,q'} . \end{aligned} \tag{S13}$$

Therefore, $\mathbf{v}$ and $\mathbf{u}$ can be calculated by solving the Maxwell equations (S6,S7) and Eqs. (S10,S11) by setting the electric and magnetic current sources of Eqs. (S8, S9) and Eqs. (S12,S13), respectively. They can be solved by finite-difference methods. Note that the electric and magnetic current sources depend on the forward and adjoint fields and the objective function.

Finally, after $\mathbf{v}$ and $\mathbf{u}$ are determined, the objective function derivative is found as

$$\frac{dT}{dp} = \frac{\partial T}{\partial p} - \sum_q \mathbf{v}_{E,q}^T \frac{\partial \mathbf{R}_{E,q}}{\partial p} - \sum_q \mathbf{v}_{H,q}^T \frac{\partial \mathbf{R}_{H,q}}{\partial p} - \sum_q \mathbf{u}_{E,q}^\dagger \frac{\partial \mathbf{R}_{E,q}^*}{\partial p} - \sum_q \mathbf{u}_{H,q}^\dagger \frac{\partial \mathbf{R}_{H,q}^*}{\partial p}, \tag{S14}$$

which can be quickly evaluated for all the parameters in a set $\mathbf{p}$ without solving additional equations.

Up to now, we have derived the general theory of adjoint-simulation based inverse design algorithm. In practical designs, the currents and derivatives can be simplified based on the type of material and form of objective function. For example, if we consider the most common case where all materials are reciprocal with symmetric permittivity and permeability, and we only have the electric field induced electric current source such that $\varepsilon = \varepsilon^T$, $\mu = \mu^T$, $\partial \mathbf{K}_{q'}/\partial \mathbf{E}_q = \partial \mathbf{K}_{q'}/\partial \mathbf{H}_q = \partial \mathbf{K}_{q'}^*/\partial \mathbf{E}_q = \partial \mathbf{K}_{q'}^*/\partial \mathbf{H}_q = \partial \mathbf{J}_{q'}/\partial \mathbf{H}_q = \partial \mathbf{J}_{q'}^*/\partial \mathbf{H}_q = 0$. Then, the current sources become

$$\mathbf{J}_{v,q} = \left(\frac{\partial T}{\partial \mathbf{E}_q}\right)^T + \sum_{q'} \left(\frac{\partial \mathbf{J}_{q'}}{\partial \mathbf{E}_q}\right)^T \mathbf{v}_{E,q'} + \sum_{q'} \left(\frac{\partial \mathbf{J}_{q'}^*}{\partial \mathbf{E}_q}\right)^T \mathbf{u}_{E,q'}^*, \tag{S15}$$

$$\mathbf{K}_{v,q} = -\left(\frac{\partial T}{\partial \mathbf{H}_q}\right)^T, \tag{S16}$$

$$\mathbf{J}_{u,q} = \left(\frac{\partial T^*}{\partial \mathbf{E}_q}\right)^T + \sum_{q'} \left(\frac{\partial \mathbf{J}_{q'}}{\partial \mathbf{E}_q}\right)^T \mathbf{u}_{E,q'} + \sum_{q'} \left(\frac{\partial \mathbf{J}_{q'}^*}{\partial \mathbf{E}_q}\right)^T \mathbf{v}_{E,q'}^*, \tag{S17}$$

$$\mathbf{K}_{u,q} = -\left(\frac{\partial T^*}{\partial \mathbf{H}_q}\right)^T. \tag{S18}$$

In the case when μ and $\mathbf{K}$ are independent on p, the derivative is expressed as

$$\frac{dT}{dp} = \frac{\partial T}{\partial p} - \sum_q \mathbf{v}_{E,q}^T \left[i\omega_q \frac{\partial \varepsilon_{q'}}{\partial p} \mathbf{E}_q - \frac{\partial \mathbf{J}_q}{\partial p}\right] - \sum_q \mathbf{u}_{E,q}^\dagger \left[-i\omega_q \frac{\partial \varepsilon_q^*}{\partial p} \mathbf{E}_q^* - \frac{\partial \mathbf{J}_q^*}{\partial p}\right]. \tag{S19}$$

For a real-valued objective function, when $T = T^*$, we have $\mathbf{u} = \mathbf{v}$, and accordingly the expression in Eq. (S19) is also real-valued and becomes

$$\frac{dT}{dp} = \frac{\partial T}{\partial p} - 2\mathrm{Re}\left[\sum_q \mathbf{v}_{E,q}^T \left(i\omega_q \frac{\partial \varepsilon_q}{\partial p} \mathbf{E}_q - \frac{\partial \mathbf{J}_q}{\partial p}\right)\right]. \tag{S20}$$

S1.2. Scattering into Specific Modes

We now consider the linear optical case with a single frequency $\omega_q = \omega$. For optimization of far-field scattering outward the surface $\mathbf{\Omega}$, we define the objective function $T = a$ as a complex

amplitude for a particular wave or mode with a field $(\mathbf{E}_f, \mathbf{H}_f)$, which can be determined as

$$a = \frac{1}{N} \iint_{\Omega} \mathbf{n} \cdot [\mathbf{E} \times \mathbf{H}_b - \mathbf{E}_b \times \mathbf{H}] \, d\Omega \,, \tag{S21}$$

where $\mathbf{n} = -\mathbf{n}_{in}$ is the unit normal vector outward the surface. The normalisation coefficient is $N = \iint_{\Omega} \mathbf{n} \cdot [\mathbf{E}_f \times \mathbf{H}_b - \mathbf{E}_b \times \mathbf{H}_f] \, d\Omega$, where $(\mathbf{E}_b, \mathbf{H}_b)$ is a direction-reversed wave. Note that this target output wave or mode could be different from the input ones defined by $(\mathbf{E}_{q,in}, \mathbf{H}_{q,in})$. These modes can be extremely general; for example they may be plane wave modes, various kinds of Gaussian beam modes, or waveguide modes, etc.

According to the vector product rules of $\mathbf{a} \cdot (\mathbf{b} \times \mathbf{c}) = \mathbf{b} \cdot (\mathbf{c} \times \mathbf{a}) = \mathbf{c} \cdot (\mathbf{a} \times \mathbf{b})$, $\mathbf{a} \times \mathbf{b} = -\mathbf{b} \times \mathbf{a}$, and $\mathbf{a} \cdot \mathbf{b} = \mathbf{a}^T \cdot \mathbf{b}$, we can transform the objective function into

$$T = a = \frac{1}{N} \iint_{\Omega} - \left[(\mathbf{n} \times \mathbf{H}_b)^T \mathbf{E} + (\mathbf{n} \times \mathbf{E}_b)^T \mathbf{H} \right] d\Omega \,, \tag{S22}$$

Then, the sources in Eq. (S15) and Eq. (S16) are surface currents on $\mathbf{\Omega}$,

$$\mathbf{J}_{v,q} = \delta(\mathbf{r} - \mathbf{\Omega}) \left(-\mathbf{n} \times \frac{\mathbf{H}_b}{N} \right) , \tag{S23}$$

$$\mathbf{K}_{v,q} = \delta(\mathbf{r} - \mathbf{\Omega}) \left(\mathbf{n} \times \frac{\mathbf{E}_b}{N} \right) . \tag{S24}$$

According to the principle of equivalence [S10], the resulting fields $\mathbf{v}_\mathbf{E}$ inside $\mathbf{\Omega}$ are equal to the fields induced by an incident wave of $(\mathbf{E}_b/N, \mathbf{H}_b/N)$ on $\mathbf{\Omega}$. The currents for generating fields $\mathbf{u}_\mathbf{E}$ will be zero as $\partial T^*/\partial \mathbf{E} = \partial T^*/\partial \mathbf{H} = 0$.

Finally, the objective function derivative is found according to Eq. (S19),

$$\frac{dT}{dp} = \frac{da}{dp} = -i\omega \mathbf{v}_E^T \frac{\partial \varepsilon}{\partial p} \mathbf{E}, \tag{S25}$$

In the case of the objective function defined as the wave intensity, $T = aa^* = |a|^2$, it is a real-valued function of a and a^*, and so the derivative will be

$$\frac{dT}{dp} = \frac{d|a|^2}{d\xi} = 2\mathrm{Re} \left[-i\omega a^* \mathbf{v}_E^T \frac{\partial \varepsilon}{\partial p} \mathbf{E} \right] . \tag{S26}$$

In the main manuscript, the optimization parameter $p = \xi(\mathbf{r})$ is defining the permittivity at each position of the design space as

$$\varepsilon(\mathbf{r}) = \varepsilon_c + \xi(\mathbf{r})(\varepsilon_d - \varepsilon_c), \; 0 \leq \xi(\mathbf{r}) \leq 1, \tag{S27}$$

where ε_d and ε_c are the permittivity for the patterned and cladding materials, respectively. Then the derivative is

$$\frac{dT}{dp} = 2\,\mathrm{Re} \left[-i\omega a^* \mathbf{v}_E^T (\varepsilon_d - \varepsilon_c) \mathbf{E} \right] . \tag{S28}$$

S1.3. Adjoints and Gradients for Optimization of SFG Processes

We consider the waves with $\omega_3 = \omega_1 + \omega_2$ in presence of quadratic nonlinearity. In the low-depletion regime, the wave evolutions at ω_1 and ω_2 are linear and their source of forward simulation are the input waves $(\mathbf{E}_{1,in}, \mathbf{H}_{1,in})$ and $(\mathbf{E}_{2,in}, \mathbf{H}_{2,in})$, and in our work are plane waves. The current source of forward simulation at the sum frequency ω_3 is

$$J_{3,i}(\mathbf{r}) = -i\omega_3\varepsilon_0\xi(\mathbf{r})\sum_{jk}\chi^{(2)}_{ijk}E_{1,j}(\mathbf{r})E_{2,k}(\mathbf{r}). \tag{S29}$$

and $\mathbf{K}_3 = 0$.

The target is to optimize the sum frequency generation for a maximum radiation to a particular diffraction order, we consider an objective function of $T = a_3$ where

$$a_3 = \frac{1}{N_3}\iint_\Omega \mathbf{n}\cdot[\mathbf{E}_3 \times \mathbf{H}_{3,b} - \mathbf{E}_{3,b}\times\mathbf{H}_3]\,d\Omega\,. \tag{S30}$$

From our previous result and using Eq. (S6), the required adjoint fields at ω_3 are the solution to the equation with sources whose input wave is $(\mathbf{E}_{3,b}/N_3, \mathbf{H}_{3,b}/N_3)$ on $\boldsymbol{\Omega}$. Meanwhile, the current sources of adjoint simulations at $\omega_{1,2}$ are

$$\mathbf{J}_{v,1} = \mathbf{L}_1^T\mathbf{v}_{E,3}\,, \tag{S31}$$

$$\mathbf{J}_{v,2} = \mathbf{L}_2^T\mathbf{v}_{E,3}\,, \tag{S32}$$

where $\mathbf{L}_1$ and $\mathbf{L}_2$ are 3×3 tensors whose elements are

$$L_{1,ij} = -i\varepsilon_0\omega_3\xi(\mathbf{r})\sum_k\chi^{(2)}_{ijk}E_{2,k}\,, \tag{S33}$$

$$L_{2,ik} = -i\varepsilon_0\omega_3\xi(\mathbf{r})\sum_j\chi^{(2)}_{ijk}E_{1,j}\,. \tag{S34}$$

The magnetic currents are $\mathbf{K}_{v,1} = \mathbf{K}_{v,2} = 0$.

Finally, the derivative is

$$\frac{dT}{d\xi} = \frac{da_3}{d\xi} = -i\varepsilon_0\omega_3\sum_{ijk}\chi^{(2)}_{ijk}E_{1,j}E_{2,k}\mathrm{v}_{E,3,i} - i\sum_q\omega_q\mathbf{v}^T_{E,q}(\varepsilon_{q,d}-\varepsilon_{q,c})\mathbf{E}_q\,. \tag{S35}$$

For an objective function defined as the sum-frequency intensity of a selected spatial mode, $T = a_3a_3^* = |a_3|^2$, the derivative is found as

$$\frac{dT}{d\xi} = \frac{d|a_3|^2}{d\xi} = \mathrm{Re}\left\{a_3^*\left[-2i\varepsilon_0\omega_3\sum_{ijk}\chi^{(2)}_{ijk}E_{1,j}E_{2,k}\mathrm{v}_{E,3,i} - 2i\sum_q\omega_q\mathbf{v}^T_{E,q}(\varepsilon_{q,d}-\varepsilon_{q,c})\mathbf{E}_q\right]\right\}. \tag{S36}$$

S1.4. Re-derivation of Maximum Second Harmonic Flux Gradients

We now determine the adjoint gradients that were derived in the works [S7, S8], and thus demonstrate the validity of our approach, with the corresponding objective function defined as

$$T = -\text{Re}\left(\int \mathbf{J}_2^* \cdot \mathbf{E}_2 d\mathbf{r}\right) = -\frac{1}{2}\left[\int \mathbf{J}_2^\dagger \mathbf{E}_2 d\mathbf{r} + \int \mathbf{E}_2^\dagger \mathbf{J}_2 d\mathbf{r}\right] . \tag{S37}$$

Before calculating the current sources for the adjoint simulation, let's calculate the derivative $\mathbf{L} = \partial \mathbf{J}_2/\partial \mathbf{E}_1$, which is a 3×3 matrix with the element (i, k) defined as

$$L_{ik} = \frac{\partial J_{2,i}}{\partial E_{1,k}} = \frac{\partial\left[-i\varepsilon_0\omega_2 p \sum_{jk} \chi_{ijk}^{(2)} E_{1,j} E_{1,k}\right]}{\partial E_{1,k}} = -2i\varepsilon_0\omega_2 p \sum_j \chi_{ijk}^{(2)} E_{1,j} \, . \tag{S38}$$

Then, we obtain the current sources for the adjoint simulations as

$$\mathbf{J}_{v,1} = -0.5\mathbf{L}^T \mathbf{E}_2^* + \mathbf{L}^T \mathbf{v}_{E,2} \, , \tag{S39}$$

$$\mathbf{J}_{v,2} = -0.5\mathbf{J}_2^* \, , \tag{S40}$$

and $\mathbf{K}_{v,1} = \mathbf{K}_{v,2} = 0$.

Finally, the objective function derivative is found using Eq. (S20).

$$\frac{dT}{dp} = \text{Re}\left[i\varepsilon_0\omega_2 \sum_{ijk} \chi_{ijk}^{(2)} E_{1,j} E_{1,k}\left(0.5E_{2,i}^* - 2\text{v}_{E,2,i}\right) - 2i\sum_q \omega_q \mathbf{v}_{E,q}^T(\varepsilon_{q,d} - \varepsilon_{q,c})\mathbf{E}_q\right] . \tag{S41}$$

When the target is to optimize the second harmonic for a maximum radiation to a particular diffraction order with electromegnetic field $(\mathbf{E}_{2,f}, \mathbf{H}_{2,f})$, we consider an objective function $T = s_2 s_2^*$ where

$$s_2 = \frac{1}{N_2}\iint_\Omega \mathbf{n} \cdot \left[\mathbf{E}_2 \times \mathbf{H}_{2,b} - \mathbf{E}_{2,b} \times \mathbf{H}_2\right] d\Omega \, . \tag{S42}$$

Similar to the linear case, the source of adjoint simulation at ω_2 is the input wave $(\mathbf{E}_{2,b}/N_2, \mathbf{H}_{2,b}/N_2)$ on $\mathbf{\Omega}$. The current source of adjoint simulation at ω_1 is

$$\mathbf{J}_{v,1} = \mathbf{L}^T \mathbf{v}_{E,2} \, , \tag{S43}$$

and $\mathbf{K}_{v,1} = 0$. Now we use Eq. (S20) and find

$$\frac{dT}{dp} = \text{Re}\left\{s_2^*\left[-2i\varepsilon_0\omega_2 \sum_{ijk} \chi_{ijk}^{(2)} E_{1,j} E_{1,k} \text{v}_{E,2,i} - 2i\sum_q \omega_q \mathbf{v}_{E,q}^T(\varepsilon_{q,d} - \varepsilon_{q,c})\mathbf{E}_q\right]\right\} . \tag{S44}$$

Additionally, the linear refractive index is also parametrized as

$$\varepsilon_q(\mathbf{r}) = \varepsilon_{q,c} + p(\varepsilon_{q,d} - \varepsilon_{q,c}) \, . \tag{S45}$$

S2. NONLINEAR POLARISATION OF InGaP

The nonlinear polarisation for (100)-oriented InGaP has the following expression

$$\begin{pmatrix} P_3^x \\ P_3^y \\ P_3^z \end{pmatrix} = \varepsilon_0 \chi^{(2)} \begin{pmatrix} E_1^z E_2^z - E_1^y E_2^y \\ -E_1^x E_2^y - E_1^y E_2^x \\ E_1^x E_2^z + E_1^z E_2^x \end{pmatrix}. \tag{S46}$$

We assume a value of $\chi^{(2)} = 110 \times 10^{-12}\,\mathrm{m\,V^{-1}}$, and is used throughout the work for optimisation and simulations.

S3. NONLINEAR SCATTERING MATRIX

We first describe the scattering matrix of a plain unpatterned film of nonlinear material. Then, we provide explicit values of the nonlinear scattering matrices of our metasurfaces and discuss the implications. The explicit form of the nonlinear scattering matrix is

$$\begin{pmatrix} E_3^H \\ E_3^V \end{pmatrix} = \underbrace{\begin{pmatrix} a_M^{HH} & a_M^{HV} \\ a_M^{VH} & a_M^{VV} \end{pmatrix}}_{M} \begin{pmatrix} E_1^H \\ E_1^V \end{pmatrix}. \tag{S47}$$

where an element a_M^{HH} is the complex nonlinear scattering coefficient that describes the transformation of $|H\rangle$ polarised signal into $|H\rangle$ polarised SFG, for a fixed pump polarisation ($|V\rangle$ in this case). Other elements are described similarly.

S3.1. Plain Unpatterned Film

For plain unpatterned films, there are no higher order diffraction orders, so only the zeroth order scattering exists. We can analytically provide the M_0 matrix for a fixed pump polarisation in the y-direction consistent with the orientation in the optimisations.

$$\mathrm{M}_0 = \begin{pmatrix} 0 & 1 \\ 1 & 0 \end{pmatrix} \tag{S48}$$

We note that this matrix has been normalised to unity for clarity, which will not be the case for typical nonlinear processes.

S3.2. Polarising Nonlinear Metasurface

We provide the zeroth order scattering matrix for the nonlinear polarising metasurface,

$$\mathbf{M}_0 = \begin{pmatrix} 0.0605 - 0.0036i & 0.0240 + 0.0154i \\ 0.6618 - 0.4265i & 0.2135 - 0.1036i \end{pmatrix} \tag{S49}$$

which have singular values of $s_1 = 0.8249$ and $s_2 = 0.0129$. The polarisation extinction is the ratio of the square of singular values, $s_1^2/s_2^2 \sim 4100$ in our case. Such large values indicate that the output is highly polarised, analogous to a linear polariser.

The left-singular vectors for $\mathbf{M}_0$ are

$$v_1 = \begin{pmatrix} -0.0792 - 0.0001i \\ -0.8377 + 0.5403i \end{pmatrix} \qquad v_2 = \begin{pmatrix} 0.4854 + 0.8707i \\ -0.0700 - 0.0371i \end{pmatrix} \tag{S50}$$

The transmission efficiency of an unpolarised state into v_1 and v_2 are given by s_1^2 and s_2^2 respectively. We see that v_1 is very close to being the desired $|V\rangle$ state, and v_2 is very close to the $|H\rangle$ state. Thus, with the large polarisation extinction, the metasurface can be considered to be a nonlinear $|V\rangle$ polariser.

S3.3. Polarization Independent Nonlinear Metasurface

We provide the zeroth order scattering matrix for the polarization independent metasurface,

$$\mathbf{M}_0 = \begin{pmatrix} 0.0130 + 0.0305i & 0.0546 - 0.0929i \\ 0.0480 - 0.0884i & 0.0425 + 0.0049i \end{pmatrix} \tag{S51}$$

which have singular values of 0.1183 and 0.1033, with $s_1^2/s_2^2 = 1.3115$. The ratio between singular values is reasonably near unity, which is reflected in the near equal SFG conversion efficiencies analysed in the $|H\rangle$ and $|V\rangle$ states (Fig. 4(e,f)).

The eigenvectors of $\mathbf{M}_0$ are

$$\mathbf{u}_1 = \begin{pmatrix} 0.7770 + 0.0000i \\ -0.6275 + 0.0496i \end{pmatrix} \mathbf{u}_2 = \begin{pmatrix} 0.6548 - 0.0295i \\ 0.7553 + 0.0000i \end{pmatrix} \tag{S52}$$

The eigenvectors are close to linear polarizations and have a phase difference of approximately 3.06 radians or 0.975π for the SFG output, and the optic axis will be aligned with these eigenvectors. We plot the output SFG polarization associated with various input signal polarizations

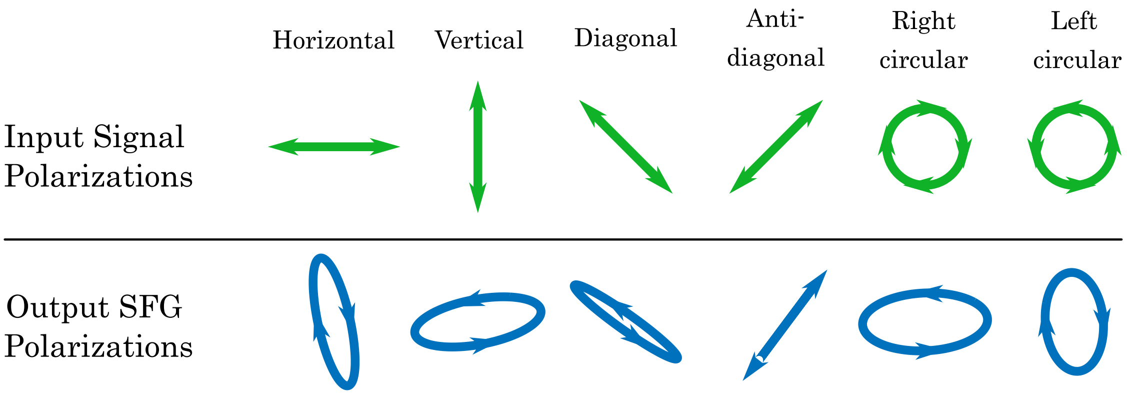

Figure S1. Select input signal polarizations at λ_1 and their corresponding output SFG polarizations after passing through the nonlinear metasurface. The amplitudes have been normalized. The pump polarization is vertical.

(Fig. S1) for the fixed pump polarization. The eigen-polarizations are close to the diagonal and anti-diagonal, consistent with the eigenvectors calculated for M_0. A property of unitary matrices is that their eigenvectors are orthogonal. To quantify the orthogonality of the eigenvectors, we calculate their modulus squared inner product,

$$|\langle \mathbf{u}_1|\mathbf{u}_2\rangle|^2 = 0.0049\,. \tag{S53}$$

The value is close to zero which suggests that the eigenvectors are almost orthogonal, and implies that M_0 is very nearly unitary.

S4. DIFFRACTION ORDER RATIO

In this section, we provide the numerical values of the diffraction efficiencies for the bar plots shown in the main manuscript.

S4.1. Polarising Nonlinear Metasurface

The zeroth order comprises more than 75% of the total SFG light from an unpolarised signal input, indicating a strong suppression of higher propagating modes (Table S1).

Diffraction Order (m,n)	Total Conversion Efficiency ($\mathrm{cm}^2\,\mathrm{GW}^{-1}$)
(0,0)	0.2722
(-1,0)	0.0090
(1,0)	0.0238
(0,-1)	0.0275
(0,1)	0.0232

Table S1. Table of conversion efficiencies for each SFG diffraction order for the nonlinear polarising metasurface.

S4.2. Nonlinear Polarization Independent Metasurface

The zeroth order comprises around 50% of the total SFG light from an unpolarised signal input, indicating a reasonably strong suppression of higher propagating modes (Table S2).

Diffraction Order (m,n)	Total Conversion Efficiency ($\mathrm{cm}^2\,\mathrm{GW}^{-1}$)
(0,0)	0.0113
(-1,0)	0.0035
(1,0)	0.0014
(0,-1)	0.0032
(0,1)	0.0046

Table S2. Table of conversion efficiencies for each SFG diffraction order for the nonlinear polarization independent metasurface.

S5. LINEAR TRANSMISSION SIMULATIONS

For high efficiency of SFG to occur, we expect resonances to be present in the metasurface at $\lambda_1, \lambda_2, \lambda_3$. We can gauge the strength of these resonances by inspecting the linear transmission of the metasurfaces, and estimating their quality factors. Qualitatively, at these resonant regimes, fields are concentrated within the nonlinear material of the metasurface, which are also the regions with largest induced nonlinear current.

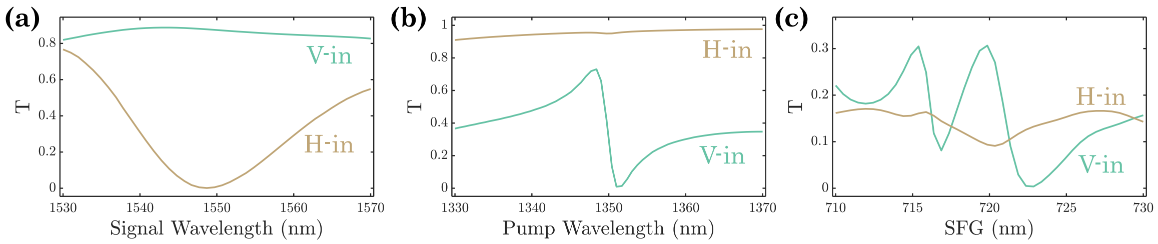

Figure S2. Linear transmission spectra of the nonlinear polarising metasurface for $|V\rangle$ and $|H\rangle$ polarisations, which are centred at (a) $\lambda_1 = 1550\,\mathrm{nm}$ (signal), (b) $\lambda_2 = 1350\,\mathrm{nm}$ (pump), (c) $\lambda_3 = 720\,\mathrm{nm}$ (SFG). See Section 3.1 in main article for relevant discussions.

S5.1. Transmission Spectra

We show the simulated linear transmission spectra for $|H\rangle$ and $|V\rangle$ incident polarisations. We centre each spectra in a range around the input pump and signal wavelengths of $1350\,\mathrm{nm}$ and $1550\,\mathrm{nm}$. The SFG wavelength is centred at $720\,\mathrm{nm}$.

The first example is of the polarising nonlinear metasurface (Fig. S2). See Section 3.1 in main article for relevant discussions. In Fig S2(a), there is a broad resonance only for $|H\rangle$ polarisation, while in Fig S2(b), only $|V\rangle$ polarisation produces a resonance. At the SFG wavelength, there is a strong resonance only for $|V\rangle$ polarisation. This is consistent with our design, where the pump is $|V\rangle$ polarised and we aim to polarise the signal input into the $|V\rangle$ state at the SFG output.

The second example is of the nonlinear polarization independent metasurface (Fig. S3). See Section 3.2 in main article for relevant discussions. We observe that in Fig. S3(a), resonances for both $|V\rangle$ and $|H\rangle$ polarisations appear at nearly the same wavelength. This is the crucial aspect in enhancing SFG conversion efficiency near equally for every incoming signal polarisation. Simultaneously, with the pump input being fixed for $|V\rangle$ polarisation, this results in only $|V\rangle$ polarisation actually producing a resonance (Fig. S3(b)). At the SFG wavelength (Fig. S3(c), $|V\rangle$ polarisation is more strongly resonant than $|H\rangle$ polarisation.

S5.2. Field Distributions

The polarisation of the pump ($\lambda_2 = 1350\,\mathrm{nm}$) input is fixed at $|V\rangle$ throughout. The electric fields are enhanced and distributed evenly across the whole device (Fig. S4). For the nonlinear polarising metasurface (Fig. S5), only an input signal ($\lambda_1 = 1550\,\mathrm{nm}$) $|H\rangle$ polarisation produces

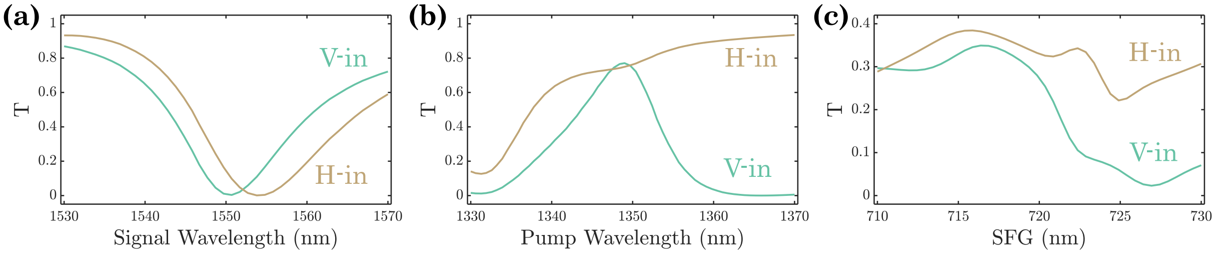

Figure S3. Linear transmission spectra of the nonlinear polarization independent metasurface for $|V\rangle$ and $|H\rangle$ polarisations, which are centred at (a) $\lambda_1 = 1550\,\mathrm{nm}$ (signal), (b) $\lambda_2 = 1350\,\mathrm{nm}$ (pump), (c) $\lambda_3 = 720\,\mathrm{nm}$ (SFG). See Section 3.2 in main article for relevant discussions.

strong field enhancement. See Section 3.1 in main article for relevant discussions. Simultaneously, there is minimal field enhancement for $|V\rangle$ polarisation, which is consistent with the resonant spectra from Fig. S2(a). We then show the induced nonlinear current which is obtained from the time derivative of Eqs. (S46), and the mixing of the electric fields of the pump and signal wave. The maximum field enhancement is from incident signal $|H\rangle$ polarisation, for which then the induced current is strongly generated. The weak orthogonal resonance barely generates a current. In both cases, only regions that have nonlinear material generate current, as labelled in the figures.

For the nonlinear polarization independent metasurface (Fig. S7), and referring to section 3.2 in main article, we see strong field enhancements for the pump wavelength (Fig. S6), and near

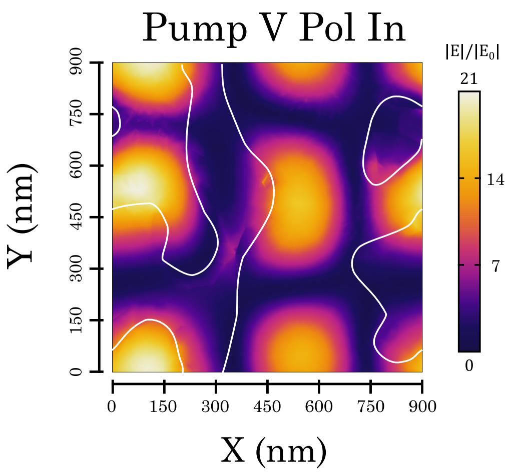

Figure S4. Nonlinear polarising metasurface. Electric field enhancement distribution for pump wavelength ($\lambda_1 = 1350\,\mathrm{nm}$) in a cross-section halfway through the nonlinear metasurface's thickness. See Section 3.1 in main article for relevant discussions.

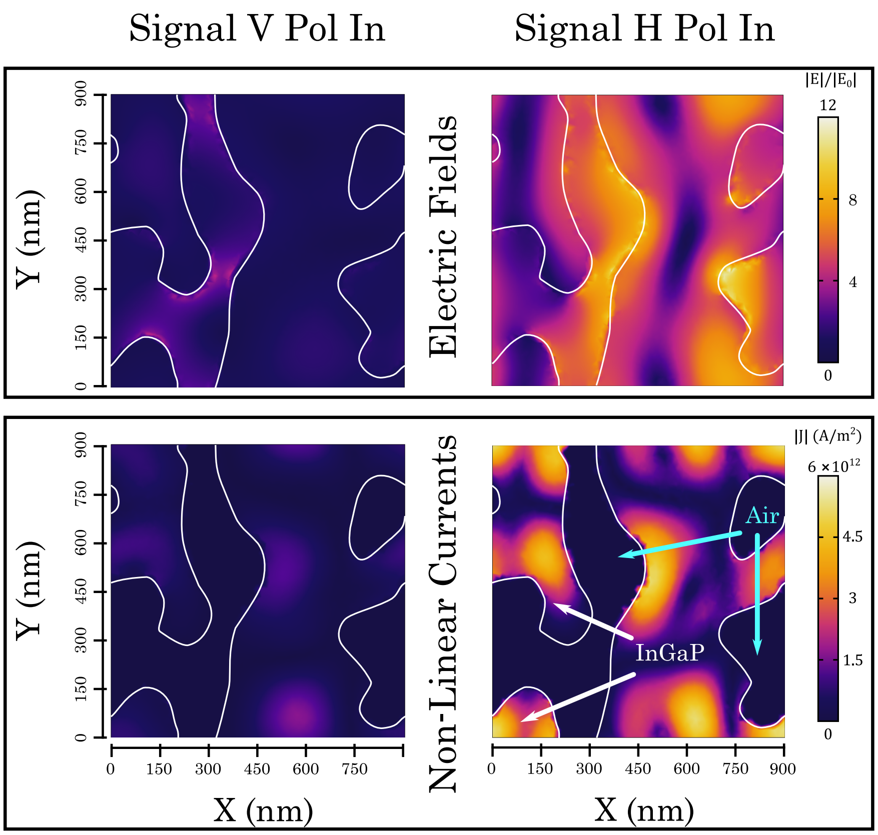

Figure S5. Nonlinear polarising metasurface. Electric field enhancement distribution (top row) and induced nonlinear current (bottom row) for signal wavelength ($\lambda_1 = 1550\,\mathrm{nm}$), with $|V\rangle$ polarisation (left column) and $|H\rangle$ polarisation (right column). Labels and arrows indicate different material regions. See Section 3.1 in main article for relevant discussions.

equal maximum electric field enhancements for both incident signal $|V\rangle$ and $|H\rangle$ polarisation. It is interesting to observe the spatial differences and similarities in which the fields are enhanced for both polarisations. As a result, the magnitude of the induced nonlinear currents are large for both polarisations.

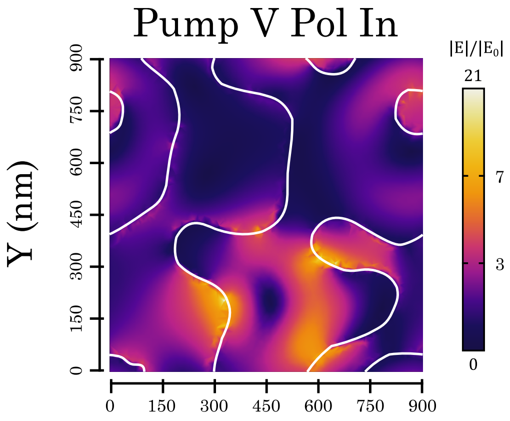

Figure S6. Nonlinear polarization independent metasurface. Electric field enhancement distribution for pump wavelength ($\lambda_1 = 1350\,\mathrm{nm}$) in a cross-section halfway through the nonlinear metasurface's thickness. See Section 3.2 in main article for relevant discussions.

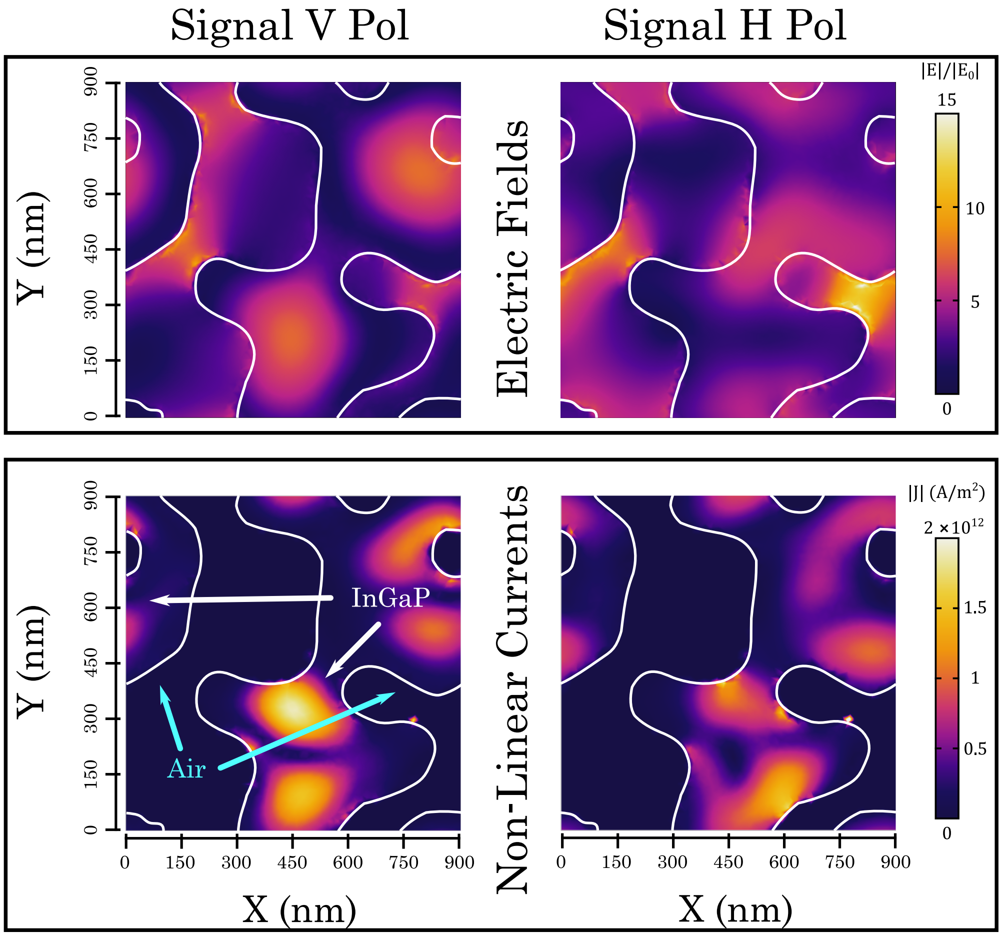

Figure S7. Nonlinear polarization independent metasurface. Electric field enhancement distribution (top row) and induced nonlinear current (bottom row) for signal wavelength ($\lambda_1 = 1550\,\mathrm{nm}$), with $|V\rangle$ polarisation (left column) and $|H\rangle$ polarisation (right column). Labels and arrows indicate different material regions. See Section 3.2 in main article for relevant discussions.

References

[S1] J. S. Jensen and O. Sigmund, Topology optimization for nano-photonics, Laser Photon. Rev. **5**, 308 (2011).

[S2] C. M. Lalau-Keraly, S. Bhargava, O. D. Miller, and E. Yablonovitch, Adjoint shape optimization applied to electromagnetic design, Opt. Express **21**, 21693 (2013).

[S3] A. C. R. Niederberger, D. A. Fattal, N. R. Gauger, S. H. Fan, and R. G. Beausoleil, Sensitivity analysis and optimization of sub-wavelength optical gratings using adjoints, Opt. Express **22**, 12971 (2014).

[S4] S. Molesky, Z. Lin, A. Y. Piggott, W. L. Jin, J. Vučković, and A. W. Rodriguez, Inverse design in nanophotonics, Nat. Photon. **12**, 659 (2018).

[S5] R. E. Christiansen, J. Michon, M. Benzaouia, O. Sigmund, and S. G. Johnson, Inverse design of nanoparticles for enhanced Raman scattering, Opt. Express **28**, 4444 (2020).

[S6] R. E. Christiansen and O. Sigmund, Inverse design in photonics by topology optimization: tutorial, J. Opt. Soc. Am. B **38**, 496 (2021).

[S7] Z. Lin, X. D. Liang, M. Loncar, S. G. Johnson, and A. W. Rodriguez, Cavity-enhanced second-harmonic generation via nonlinear-overlap optimization, Optica **3**, 233 (2016).

[S8] C. Sitawarin, W. L. Jin, Z. Lin, and A. W. Rodriguez, Inverse-designed photonic fibers and metasurfaces for nonlinear frequency conversion [invited], Phot. Res. **6**, B82 (2018).

[S9] S. A. Mann, H. Goh, and A. Alu, Inverse design of nonlinear polaritonic metasurfaces for second harmonic generation, ACS Photonics **10**, 993 (2023).

[S10] A. Oskooi and S. G. Johnson, Electromagnetic wave source conditions (2013), arXiv:1301.5366 [physics.comp-ph].
